# Treatment of femoral neck fractures: sliding hip screw or cannulated screws? A meta-analysis

**DOI:** 10.1186/s13018-020-02189-1

**Published:** 2021-01-14

**Authors:** Yutong Xia, Wendong Zhang, Zhen Zhang, Jingcheng Wang, Lianqi Yan

**Affiliations:** 1 Dalian Medical University, Dalian, 116044 Liaoning Province China; 2 Department of Orthopedics, Northern Jiangsu People’s Hospital, Yangzhou, 225001 China; 3 The second Xiangya hospital of Central South University, Changsha, Hunan 410012 China

**Keywords:** Femoral neck fracture, Sliding hip screw, Cannulated compression screw, Internal fixation, Meta-analysis

## Abstract

**Purpose:**

Femoral neck fractures are still unsolved problems nowadays; sliding hip screw (SHS) and cannulated compression screw (CCS) are the most commonly used devices. We evaluated the clinical outcomes and complications in the treatment of femoral neck fractures between SHS and CCS in this meta-analysis to find which is better.

**Methods:**

We searched PubMed, Embase, Cochrane library up to 24 August 2020 and retrieved any studies comparing sliding hip screw and cannulated compression screw in treatment of femoral neck fractures; the main outcomes and complications were extracted from the studies which were included.

**Results:**

Nine studies involving 1662 patients (828 patients in the SHS group and 834 patients in the CCS group) were included in this study. SHS had higher rate of avascular necrosis (RR = 1.30, 95% CI 1.08–1.56, *p* = 0.005), and CCS had higher rate of implant removal (RR = 0.63, 95% CI 0.43–0.93, *p* = 0.02). No significant statistical difference in non-union, implant failure, infection, replacement, mortality, orthopedic complications, non-orthopedic complications, and total revision between SHS and CCS group.

**Conclusion:**

Both devices have their pros and cons; SHS had a higher rate of avascular necrosis, and CCS had a higher rate of implant removal rate. No significant statistical difference in non-union, implant failure, infection, replacement, mortality, orthopedic complications, non-orthopedic complications, and total revision between SHS and CCS group.

## Introduction

Femoral neck fractures account for more than half percent of all hip fractures; in elderly people, they are generally caused by low energy such as falling, as for young people, they are often caused by high energy like vehicle accidents [1–3]. Femoral neck fractures will be continuously increasing in the next 30 years [4], which will make a great medical and economic burden [5–7]. The surgical methods for the treatment of femoral neck fracture are numerous, but they vary according to the patient’s age and fracture type [8–10]. American Academy of Orthopedic Surgeons (AAOS) recommends that displaced femoral neck fractures in elderly patients over 80 years of age with weak mobility should receive total hip arthroplasty or hemiarthroplasty to get the best outcomes [11]. For younger patients and undisplaced femoral neck fractures, internal fixation is the best choice. It is less invasive, can preserve the femoral head, and the hip function is better after healing [12]. However, orthopedic surgeons are often perplexed by postoperative complications of internal fixation, such as avascular necrosis, non-union, implant failure, and reoperation [13, 14]. So, we must find the most reliable implant to deal with this kind of fracture, especially for young patients, internal fixation is the first choice. According to a questionnaire study, 47% of orthopedic surgeon chose angle-fixed device (DHS with or without anti-rotation screw) to fix the fracture, while 43% of surgeon chose cannulated compress screws (CCSs) to solve the problem; these two kinds of implants are the mainstream nowadays [15]. However, there is not any consensus on which is the real gold standard.

We performed this meta-analysis and evaluated the clinical outcomes and complications in the treatment of femoral neck fractures between SHS and CCS to find which is better. This study aimed to provide reliable evidence for the internal fixation treatment of femoral neck fractures.

## Materials and methods

Our study was done according to the preferred reporting items for systematic reviews and meta-analyses (PRISMA) statement [16].

### Literature search

We searched three electronic databases (PubMed, Embase, Cochrane library) to get all articles on sliding hip screw (SHS or DHS) and cannulated screw for treating femoral neck fracture with the search terms: (sliding hip screw OR dynamic hip screw or cannulated screw) AND (femoral neck fracture OR intracapsular hip fracture) from database established up to 24 August 2020. We also did a manual examination to get the whole relevant published or under pressed articles.

### Inclusion and exclusion criteria

Abstract of all acquired and retrieved studies were examined. Studies were included if they were eligible for the requirements: (1) randomized clinical trial studies (RCTs) or controlled clinical trial studies (CCTs); (2) original literature published as full manuscripts; (3) having definite sample size, study time, race, and gender were not limited; (4) comparison of complications such as avascular necrosis, non-union, implant failure, and reoperation or non-orthopedic complications; (5) at least 1 year time to follow-up.

Studies were ineligible for this study if the following existed: (1) non-randomized trials, observational studies, biomechanical studies, and case reports; (2) undefined sample and control source, animal experiments, and non-therapeutic clinical studies; (3) non-original studies and undefined group.

### Quality assessment and data extraction

Two researchers independently appraised the cited studies’ quality in line with the Cochrane Collaboration guidelines; items in seven aspects with low, high, or unclear risk of bias were assessed.

Data extraction was carried out critically and independently by two researchers, disputes were solved by the third researcher, the following items were extracted from the included articles: name of the first author, publication year, experiment design, sample size and characteristics, interventions, follow-up time, blood loose (ml), operation time (min), treatment outcome, complications, reoperation rate, and others.

### Statistical analysis

All of the data were analyzed by Review Manager 5.4 (The Cochrane Collaboration, 2020). We used standardized mean differences (SMD) and 95% confidence intervals (CI) to express continuous data, and risk ratio (RR) with 95% CI to present dichotomous data. *p* ≤ 0.05 means statistically significant. Then, we appraised heterogeneity by Q testing and *I*^2^ statistics, if *p* ≤ 0.10 or *I*^2^ > 50%, indicating significant heterogeneity, and then we used the random-effects model to evaluate the system. On the contrary (*p* > 0.10 or *I*^2^ < 50%), the fixed-effect model was selected.

## Results

### Included articles characteristics

**Fig. 1:**
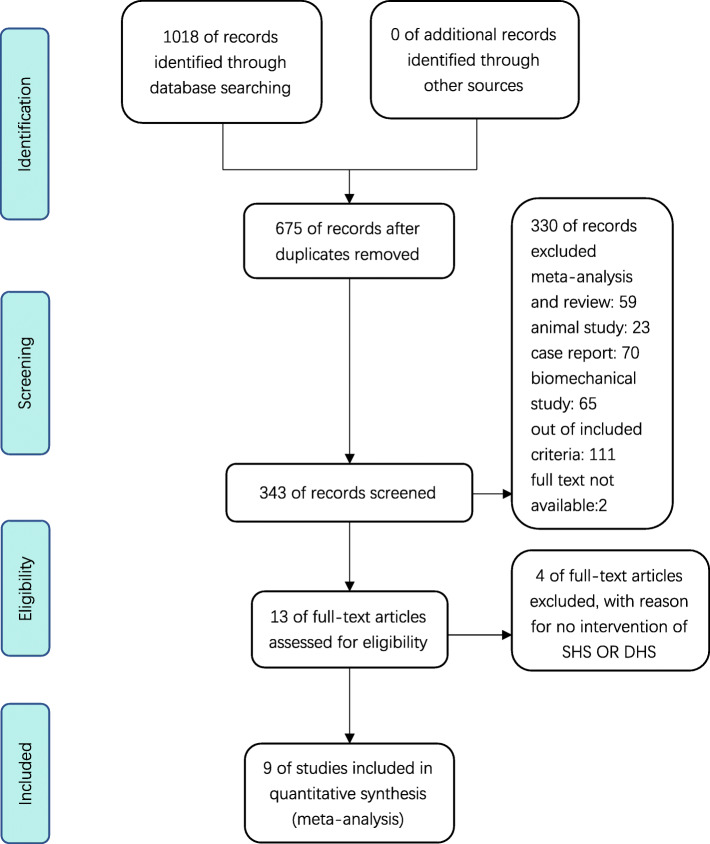
The process of selecting the included studies

We searched 3 databases and got 1018 potentially studies; after screening the articles, 9 studies [17–25] containing 1662 patients (828 patients in the SHS group and 834 patients in the CCS group) were included in this study (Fig. 1). The related characteristics were presented in Table 1.

**Table 1:** Included articles characteristics

Study/year	n (SHS/CS)	Mean age (SHS/CS)	Gender(M/F) (SHS/CS)	Intervention	Follow-up(month)
Linde 1986	40/47	76/76	(16/24)/(14/33)	SHS vs Four CCS	-
Madsen 1987	51/52	75/74	(14/37)/(11/41)	SHS vs Four CCS	24
Kuokkanen1991	17/16	60/72.5	-	SHS vs Three CCS	24
Sorensen 1992	35/38	75/76.14	(10/25)/(8/30)	DHS vs Three GCS	36
Watson 2012	30/28	77.9/76.7	(6/25)/(5/24)	DHS vs Three CCS	24
Siavashi 2015	30/28	30/28	(25/5)/(21/7)	DHS vs Three CCS	36
Gupta 2016	40/45	40.7/39.3	(23/17)/(32/13)	SHS vs Three CCS	48
FAITH 2017	542/537	72.2/72	(212/323)/(210/325)	SHS vs Three CCS	24
FAITH-2 2020	43/43	43/39.2	(30/13)/(33/10)	SHS vs Three CCS	12

### Quality assessment

**Fig. 2:**
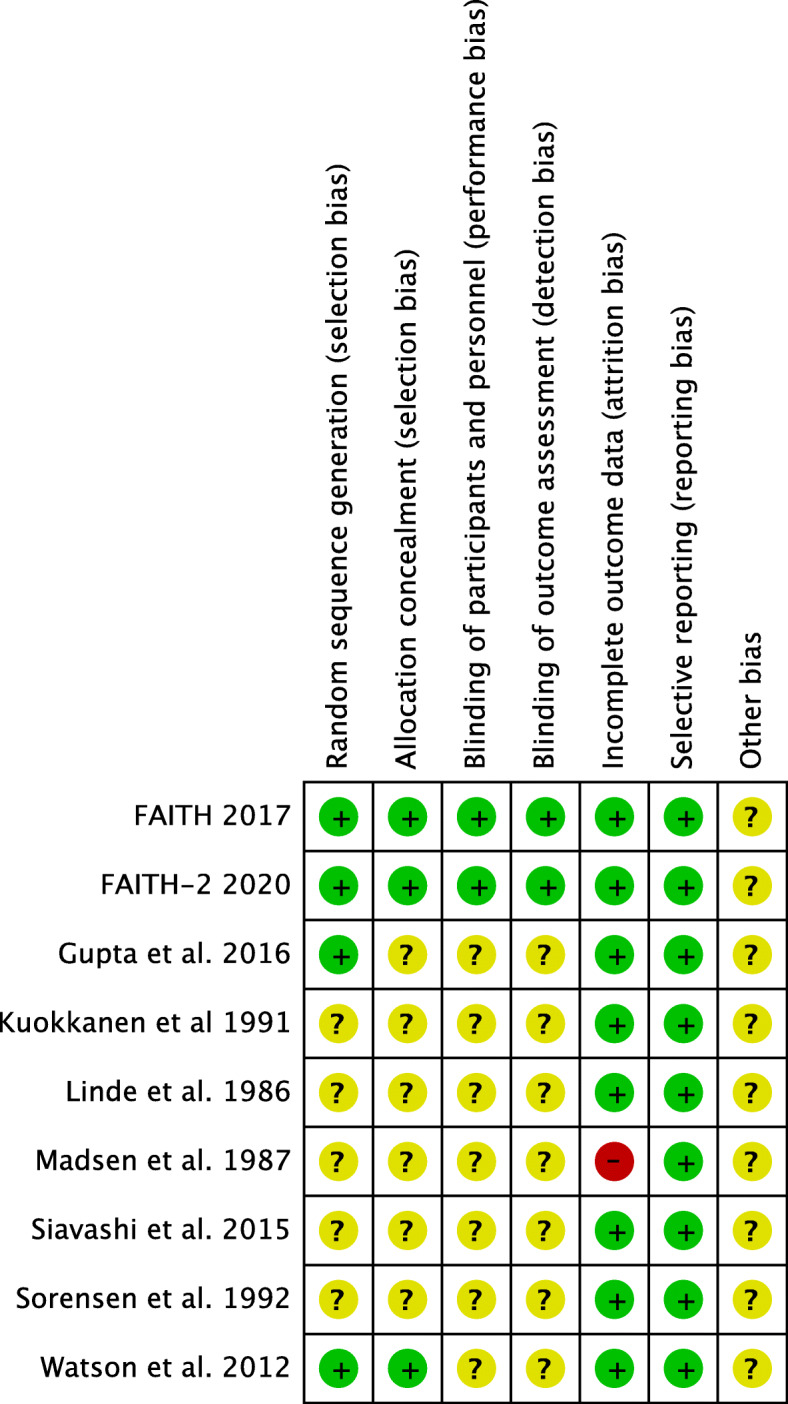
The risk bias summary of the included studies

**Fig. 3:**
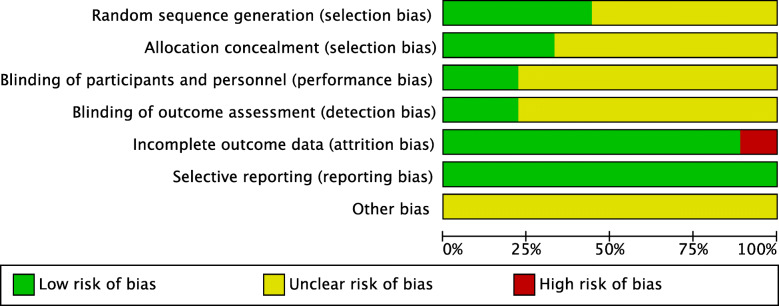
The risk of bias graph of the included studies

All the include RCT studies reported that they randomly assigned the registered patients to different implant groups, four of the nine studies reported the method of randomization [21, 23–25]. All of the studies had a low risk of bias of selective blinding. Eight trials had a low risk of bias of incomplete outcome of the data [17, 19–25]. The risk bias summary and risk of bias graph of the included studies were showed in Figs. 2 and 3, respectively.

## Results of the meta-analysis

### Avascular necrosis

**Fig. 4:**
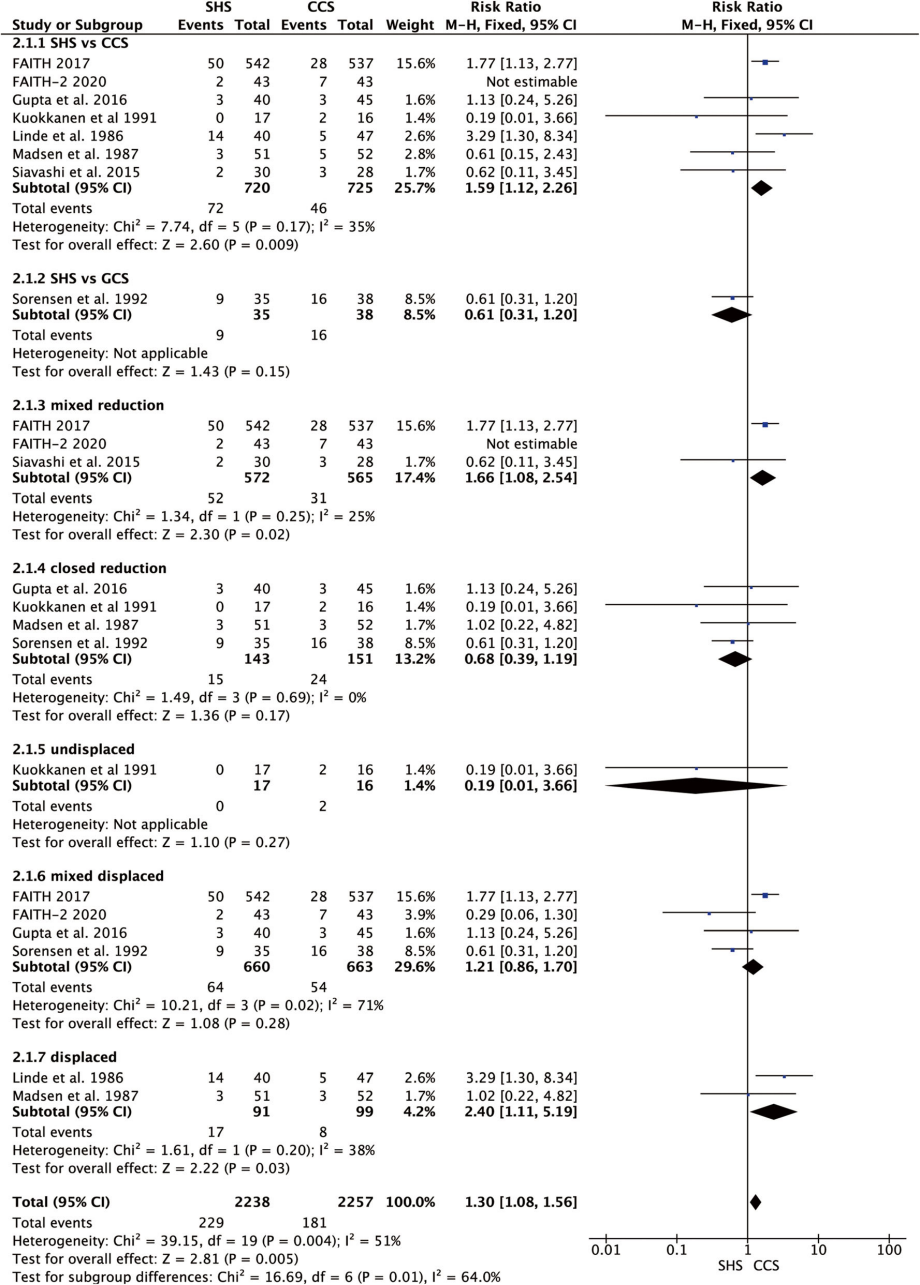
Forest plot of the comparison between SHS and CCS in avascular necrosis rate

Eight studies reported the avascular necrosis rate [17–20, 22–25]. We did the subgroup analysis according to the type of cannulated screw, the different methods of reduction (closed reduction, open reduction, or mixed-method of reduction), and the different types of femoral neck fracture (displaced, undisplaced, and mixed). A sensitivity analysis was done by excluding the FAITH-2 [25] in subgroup type of cannulated screw (*p* = 0.17, *I*^2^ = 35%) and mixed-method of reduction (*p* = 0.25, *I*^2^ = 25%), SHS showed a higher avascular necrosis rate in comparison with CCS (*p* = 0.009 and *p* = 0.02, respectively, Fig. 4). Meanwhile in subgroup displaced femoral neck fracture, they were homogenous (*p* = 0.20, *I*^2^ = 38%), and SHS also showed a higher avascular necrosis rate in comparison with CCS (RR = 2.40, 95% CI 1.11–5.49, *p* = 0.03, Fig. 4).

### Non-union

**Fig. 5:**
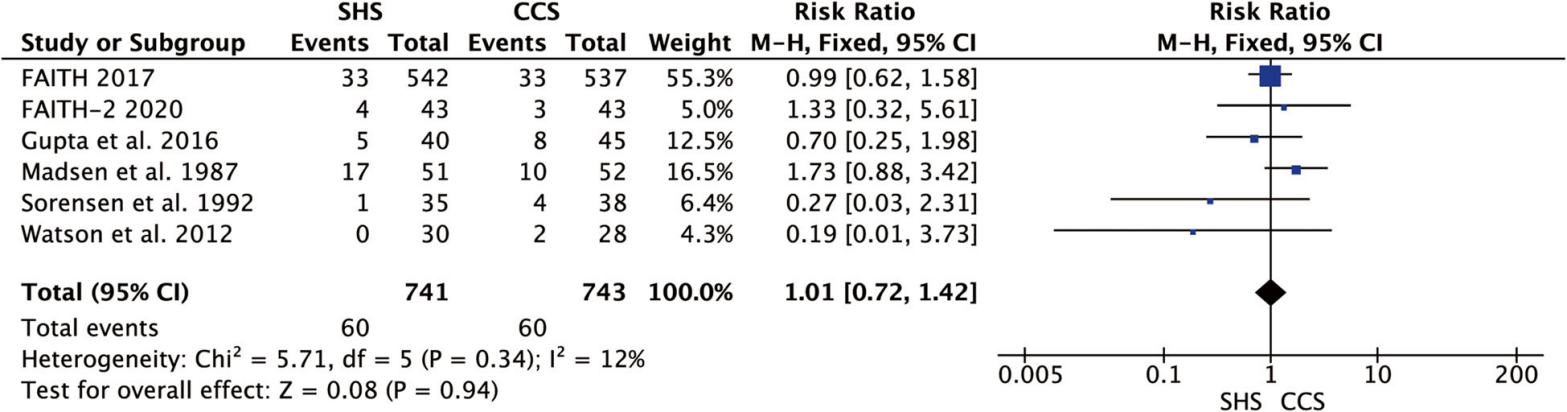
Forest plot of the comparison between SHS and CCS in non-union rate

Six studies reported the non-union rate [18, 20, 21, 23–25], and they were homogenous (*p* = 0.34, *I*^2^ = 12%). The result showed no statistical difference in the non-union rate between the two groups (RR = 1.01, 95% CI 0.72–1.42, *p* = 0.94, Fig. 5).

### Failure

**Fig. 6:**
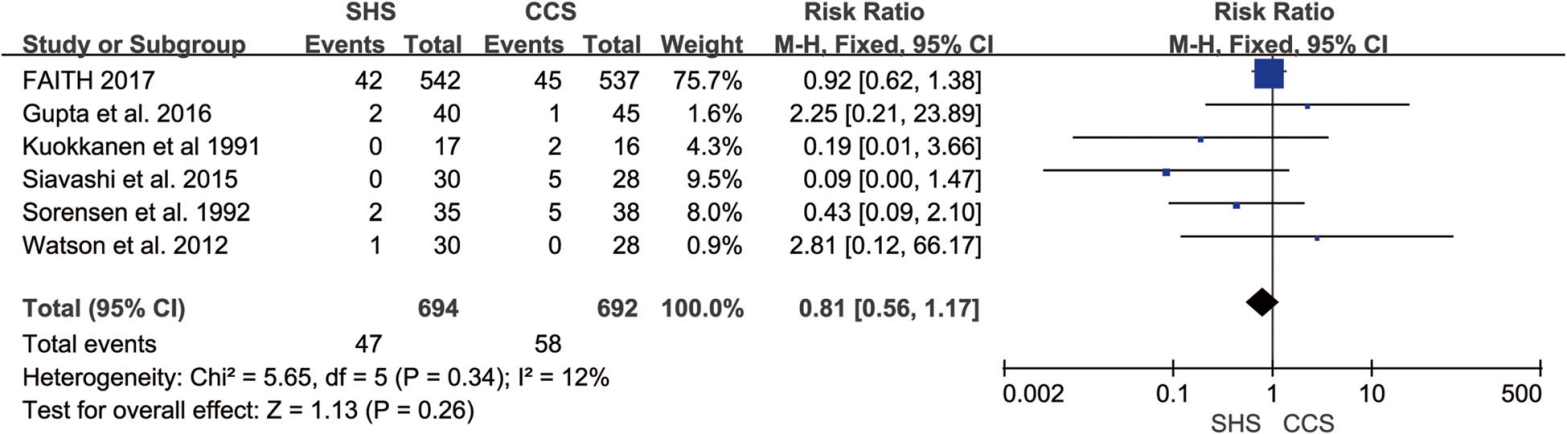
Forest plot of the comparison between SHS and CCS in failure rate

Six studies reported the failure rate [19–24]. The studies were homogenous (*p* = 0.34, *I*^2^ = 12%). The result showed not any statistical difference in failure rate between SHS and CCS (RR = 0.81, 95% CI 0.56–1.17, *p* = 0.26, Fig. 6).

### Infection

**Fig. 7:**
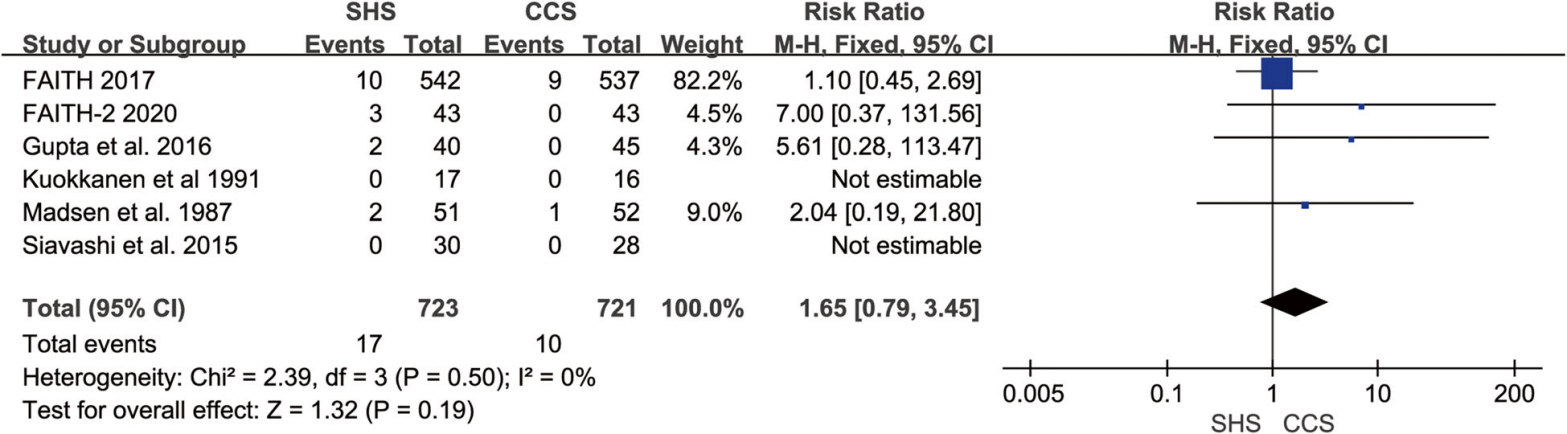
Forest plot of the comparison between SHS and CCS in infection rate

Six studies reported the infection rate [18, 19, 22–25]. The studies were homogenous (*p* = 0.50, *I*^2^ = 0%). The result presented no statistical difference in infection rate between SHS and CCS (RR = 1.65, 95% CI 0.79–3.45, *p* = 0.19, Fig. 7).

### Implant removal

**Fig. 8:**
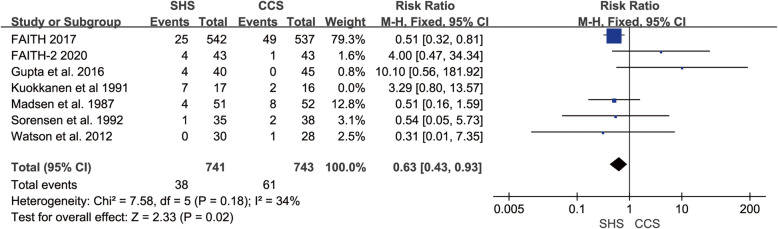
Forest plot of the comparison between SHS and CCS in implant removal

Seven studies reported the implant removal rate [18–21, 23–25]. There was heterogeneity across the seven studies (*p* = 0.04, *I*^2^ = 54%). So, the sensitivity analysis was conducted by excluding Kuokkanen et al. [19], then the remaining studies were homogeneous (*p* = 0.18, *I*^2^ = 34%). CCS shown higher implant removal rate compared to SHS (RR = 0.63, 95% CI 0.43–0.93, *p* = 0.02, Fig. 8).

### Replacement

**Fig. 9:**
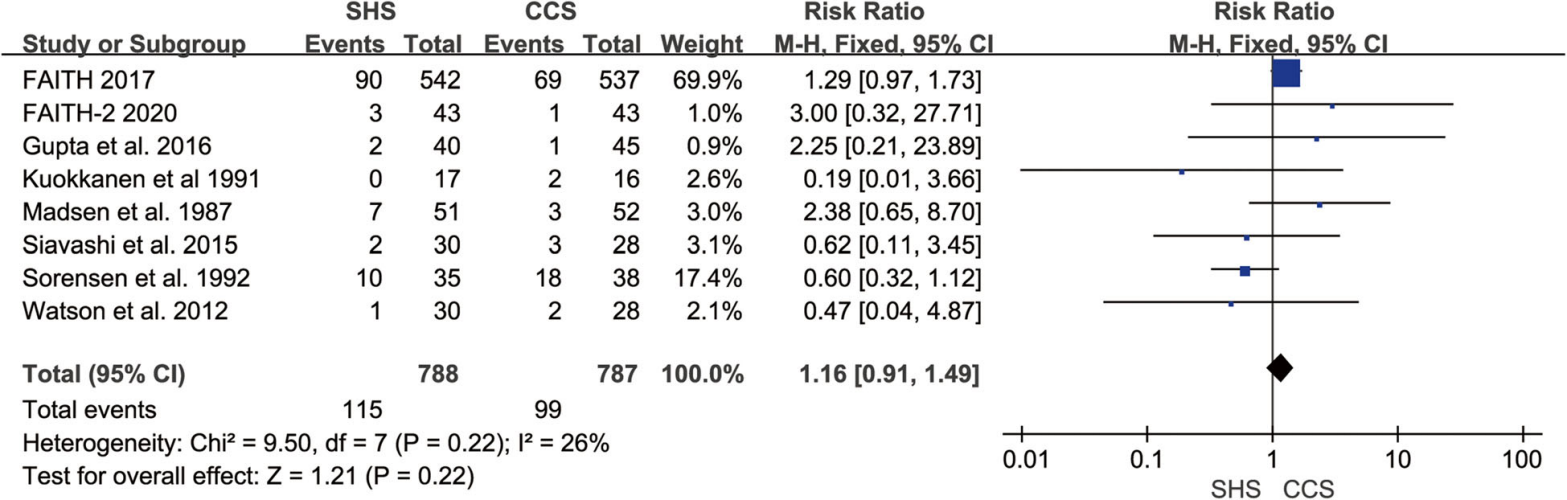
Forest plot of the comparison between SHS and CCS in the replacement rate

Eight studies reported the replacement rate [18–25]. And the studies did not have any heterogeneity (*p* = 0.22, *I*^2^ = 26%). The result indicated no statistical difference in replacement rate between the two groups (RR = 1.16, 95% CI 0.91–1.49, *p* = 0.22, Fig. 9).

### Mortality

**Fig. 10:**
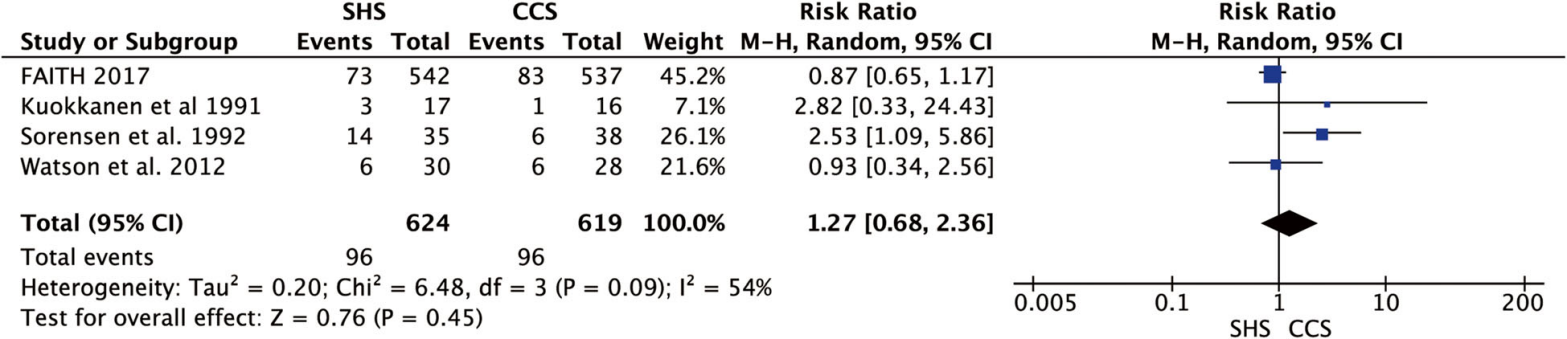
Forest plot of the comparison between SHS and CCS in mortality rate

Four studies reported the mortality rate [19–21, 24]. Only a little heterogeneity was found between the studies (*p* = 0.09, *I*^2^ = 54%), and we used the random-effect model to merge the data, and indicated no statistical difference in mortality rate between two groups (RR = 1.27, 95% CI 0.68–2.36, *p* = 0.45, Fig. 10).

### Orthopedic complications

**Fig. 11:**
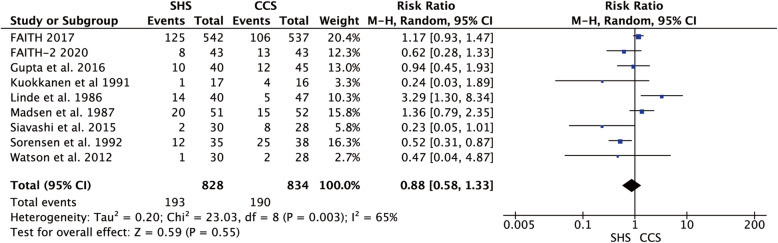
Forest plot of the comparison between SHS and CCS in orthopedic complication

All nine articles presented the orthopedic complications post-operation; there was heterogeneity among them (*p* = 0.003, *I*^2^ = 65%), so the data were merged using a random-effect model and show no statistical difference in postoperative orthopedic complications between two groups (RR = 0.88, 95% CI 0.58–1.33, *p* = 0.55, Fig. 11).

### Non-orthopedic complications

**Fig. 12:**
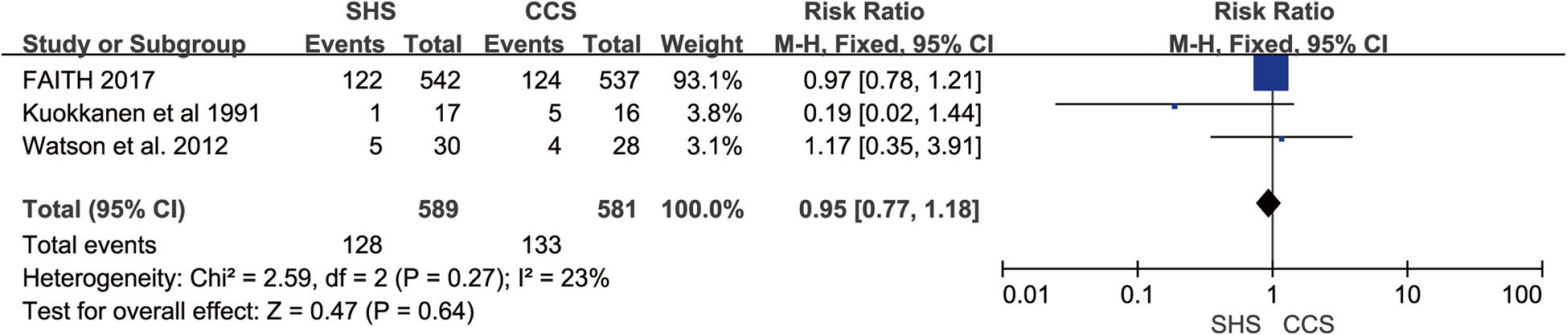
Forest plot of the comparison between SHS and CCS in non-orthopedic complication

Only three studies mentioned the non-orthopedic complications after operation [19, 21, 24], and they were homogenous (*p* = 0.27, *I*^2^ = 23%). The result showed no statistical difference in replacement rate between SHS and CCS (RR = 0.95, 95% CI 0.77–1.18, *p* = 0.64, Fig. 12).

### Total revision

**Fig. 13:**
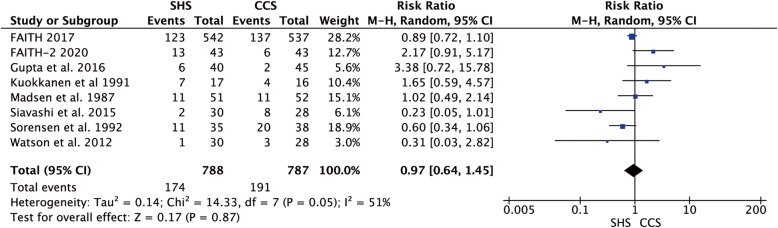
Forest plot of the comparison between SHS and CCS in total revision rate

Eight studies reported the revision rate after internal fixation of femoral neck fracture s[18–25]. We found a little heterogeneity between the studies (*p* = 0.05, *I*^2^ = 51%) and used the random-effect model to pool the data, result indicated no statistical difference in revision rate between the two groups (RR = 0.97, 95% CI 0.64–1.45, *p* = 0.87, Fig. 13).

## Discussion

Femoral neck fractures need an acute operation, accurate anatomical reduction, and stiff internal fixation. Treatment therapy of femoral neck fracture depends on the age of patients and the classification; internal fixation is the priority of young patients and also a good choice for active elderly. Arthroplasty is the ultimate choice when internal fixation failed or complications occurred [26]. SHS and CCS are the most commonly used implants nowadays, but both of them have the disadvantage.

In this meta-analysis, we can indicate that the avascular necrosis rate was higher in the SHS group, and the implant removal rate was higher in the CCS group. While no significant statistical difference between the SHS and CCS groups in terms of non-union, implant failure, infection, replacement, mortality, orthopedic complications, non-orthopedic complications, and total revision. We defined orthopedic complications like avascular necrosis, non-union, malunion, delay union, implant cut-out (penetration), implant failure, and periprosthetic fractures. Non-orthopedic complications included cardiovascular disease, gastrointestinal symptoms, pneumonia, urinary infection, deep vein thrombosis, and other diseases. Total revision referred to total arthroplasty, hemiarthroplasty, refixation, and removal of the implant.

As for avascular necrosis of the femoral head, when rotating in the lag screw of SHS or beating in the blade screw of DHS, the rotation strength may cause the femoral head displaced then influence the blood supply it. Besides, according to the subgroup analysis, the type of femoral neck fracture and the operation mode also has an impact on the blood supply of the femoral head; displaced fracture and open reduction would cause a higher rate of avascular necrosis when using SHS or DHS to deal with it compared with CCS. The Gouffon screw is a kind of partly threaded lag screw with a small diameter; the author said it was difficult to decide the length of the Gouffon screw, and changed screw led to damage to the cancellated bone of the femoral head [20]. Another reason that caused the failure is that small diameter screws cannot hold the trabecular bone strength fully [27], so we did the subgroup analysis and reduced the heterogeneity. The reduction method also influences the prognosis of a femoral neck fracture, and open reduction is associated with a high rate of complications [28]; all the studies except FAITH-2 done almost close reduction; however, in FAITH-2, open reduction was used in the majority (55.8%), which caused bias and we excluded it when analyzing avascular necrosis. Therefore, we should choose minimally invasive technology to maximize the protection of femoral head blood supply, combined with the concept of systematic treatment to shorten the length of hospital stay and speed up rehabilitation [29, 30].

Dynamic compression occurred during the healing of femoral neck fracture when using SHS or CCSs, causing a shortening of the femoral neck. CCSs would back-out from the lateral cortex make locally uncomfortable, while SHS has a telescope-like structure, the blade or lag screw back-out within the sleeve, little protrusion happened in the local place, thus the implant removal was higher in the CCS group.

Different from the published articles, we included in the latest RCT article, and we also did the subgroup analysis according to the type of femoral neck fracture, type of cannulated screws, and the operation mode to find out the factors that affect the final results, making the results more accurate. Meanwhile, the time duration post-trauma of patients in the included study was less than 72 h, and the RCT study had the highest level of evidence to minimize the risk of bias.

Both SHS and CCS have their pros and cons; so, we can combine the merit of them and design new implants with the ability such as minimally invasive, anti-rotation, and angle stable [31], making less influence on the blood supply of femoral neck and can realize instant compression. Restricting dynamic compression is still an unsolved problem.

## Limitations

First, a small number of studies were included in the meta-analysis, and some trials did not report the exact random methods that would cause a certain degree of bias. The following time of included trials was different; long-term observation would find more complications.

Second, the original data of included studies were insufficient to meet the Gaussian distribution and would make bias to the analysis; we could not analyze the Harris hip score, blood loss, and operation time, though we had the formula to calculate the mean and standard deviation.

Finally, we did not analyze the outcomes of different age and sex.

## Conclusions

This meta-analysis provides evidence that SHS has a higher rate of avascular necrosis, while CCS has a higher rate of implant removal rate. There is no difference between the two groups in non-union, failure, infection, replacement, mortality, orthopedic complications, non-orthopedic complications, and total revision.

## Data Availability

All data generated or analyzed during this study are included in this published article.

## Abbreviations

SHS: Sliding hip screw
CCS: Cannulated compression screw
AAOS: American Academy of Orthopedic surgeons
DHS: Dynamic hip screw
RCTs: Randomized clinical trial studies
CCTs: Controlled clinical trial studies
SMD: Standardized mean differences
CI: Confidence intervals
